# Estimation of wheel slip in 2WD mode for an agricultural tractor during plowing operation using an artificial neural network

**DOI:** 10.1038/s41598-023-32994-7

**Published:** 2023-04-12

**Authors:** Naji Mordi Naji Al-Dosary, Fai’z Mohammed Alnajjar, Abd El Wahed Mohammed Aboukarima

**Affiliations:** 1 Department of Agricultural Engineering, College of Food and Agriculture Sciences, King Saud University, P.O. Box 2460, Riyadh, 11451 Saudi Arabia; 2 Faculty of Mechanical Engineering, University of Aleppo, Aleppo, Syria; 3 Agricultural Research Centre, Agricultural Engineering Research Institute (AEnRI), Giza, Egypt

**Keywords:** Energy science and technology, Engineering, Mathematics and computing

## Abstract

As artificial neural networks (ANNs) have been shown to be precise and reliable in supporting the field of artificial intelligence technology, agricultural scientists have focused on employing ANN for agricultural applications. The ANN can be an effective alternative for evaluating agricultural operations. The intended aim of this investigation was to employ both ANN and multiple linear regression (MLR) to develop a model for determining the rear wheel slip of an agricultural tractor in two-wheel drive (2WD) mode during plowing operations. The output parameter of the models was tractor rear wheel slip. The training data were collected from filed experiments using chisel, moldboard, and disk plows. The plows were operated under different conditions of soil texture, plowing depth, soil moisture content, and plowing speed. All data were acquired during field experiments in two soil textures (clay and clay loam textures). The training dataset was comprised of 319 data points, while 65 data points were employed to test both ANN and MLR models estimation capability. The ANN model with a backpropagation training algorithm was created using the commercial Qnet2000 software by changing its topology and related parameters. The best ANN model possessed a topology of 7-20-1. The estimated tractor rear wheel slip using the testing dataset displayed strong agreement with measured tractor rear wheel slip with the coefficient of determination (R^2^) value of 0.9977. The results definitely illustrated that the ANN model was capable of defining the correlation between the inputs and rear wheel slip. The outcomes suggest that the established ANN model is trustworthy in predicting the tractor rear wheel slip for an agricultural tractor in 2WD mode during the tillage process compared to MLR models. This study provides a useful tool for management of tillage implements during field operations.

## Introduction

The optimum choice of soil preparation implements must be supported by economical basics derived from operating factors in each field. The tillage process requires higher power and accounts for 55–60% of total field energy consumed in agricultural production^1^. However, the crucial factors are the land area and its geography which forces the farmer to choose the manner of tillage based upon its efficiency. The second level factors are the soil texture and its moisture, the type of plow, the depth of tillage, and the plowing speed. However, slippage of the tractor wheels is significantly influenced by tillage speed, soil moisture content, and the tillage depth during the tillage process with a specified tillage implement^2^. Moreover, these factors are directly related to the required draft force, and the draft force is closely associated with the tractor drive wheel slip. Additionally, slippage of a tractor drive wheel is correspondingly associated to the load on it and its inflation pressure. In an early report, Stanley^3^ mentioned that the percentage of wheel slip of a tractor is calculated as the difference of two speeds, drive wheel speed and forward speed, and noted that a tractor wheel must grip the soil to create a pulling force. Under most conditions, tractor drive wheel slippage should range between 10 and 15 percent. This is a compromise for various soil types because usually it is impractical to add or remove additional tractor weight when working different soil types^3^. However, during plowing operations the recommended value of slippage must be approximately 8–12% and if the slippage is oversized or too low, energy waste increases^4,5^.

The prediction of tractor performance parameters is associated to values of wheel slip, which is related to soil–wheel variables^6^. However, wheel slip is one of the key variables that shows surface interaction between the tires and the roads or soils^4,5,7^. Moreover, it is an important measure for assessing the fuel consumption of a tractor during field operation^8^. Accordingly, numerous efforts have been undertaken to measure and model rear and front wheel slippages^8–12^. Additionally, measurement of the slip of tractor tires with different implements and field conditions makes the estimation of instantaneous slip exceedingly difficult. Therefore, with the advancement and increased use of soft computing methods, several studies were undertaken to develop models to predict wheel slip for 2WD or 4WD tractors. Shafaei et al.^13^ applied two techniques namely: ANN and adaptive neuro-fuzzy inference system for determining tractor wheel slip during the plowing process. They examined the effects of plowing speed with three levels (2, 4, and 6 km/h), plowing depth with three levels (10, 20, and 30 cm), and both 2WD and 4WD modes of tractor drive on tractor wheel slip.

Many studies have noted that during tillage operations, there are numerous parameters affecting tractor drive wheel slip. These parameters involve tillage speed, soil type, tractor driving wheels’ style, type of the tillage implements, tillage depth, and soil moisture content^2,6,10,14^. For example, Tayel et al.^2^ studied the influence of three intensities of moisture content of the soil (8.60, 10.35, 11.61 w/w), on tractor rear wheel slip under two stages of tillage speeds (1.79 km/h and 9.6 km/h), and three stages of tillage depths (10, 20, and 30 cm). The results revealed that decreasing the soil moisture content reduced tractor rear wheel slip. Meanwhile, increasing the tillage depth caused an increase in tractor rear wheel slip, which can be attributed to the influence of overburden pressure of the upper surface of the soil on the subsoil layers and the lessening percentage of organic matter in the soil with increasing soil depth.

Performing runs of field experiments to estimate the impact of all variables on tractor rear wheel slip is both time and cost intensive. Therefore, a comprehensive computer model that evaluates the influence of the associated variables on the rear wheel slip of a tractor during tillage process is a cost effective method to extend and coordinate discrete investigational results with integral results from limited field experiments. Hence, the goal of this investigation was to create and compare the performance of an ANN model and MLR models for predicting the rear wheel slip of a tractor in 2WD mode under tillage operation parameters of plowing speed, plowing depth, soil type, and moisture content of the soil.

## Materials and methods

### Soil investigations

An experimental field with two soil textures (i.e., clay and clay loam textures) was used to carry out the plowing tests. The first experimental site (site I) had a clay texture (20.16% silt, 30.03% sand, and 49.81% clay). The second experimental site (site II) had a clay loam texture (38.05% silt, 29.93% sand, and 32.02% clay). The moisture content and bulk density of the soil at the experimental sites were determined by acquiring four soil samples at various positions of the sites. The samples were obtained from different soil layers at 10, 15, 20 and 25 cm. The soil samples were removed from the soil layers by a cylindrical core sampler. The soil samples were immediately placed in plastic bags to keep moisture content constant during transfer to the laboratory. Each soil sample was weighted and dried in an electric oven at 105 °C for 24 h. The average soil bulk density was 1.44 g/cm^3^ and 1.41 g/cm^3^ at the site I and site II, respectively. The organic matter content was 0.94% and 1.18% at the site I and site II, respectively.

In this study, the soil texture was presented by a soil texture index symbolized by STI with dimensionless. This index combined the percentages of the clay, sand, and silt in the soil and was offered by Oskoui and Harvey^15^ as follows.

1 $$ STI = \frac{{log ({\text{Ca}}^{Si} + Sa )}}{100} $$

here *Sa* symbolized the sand percentage in the soil layers, *Si* symbolized the percentage of silt in the soil layers, and *Ca* symbolized the percentage of clay also in the soil layers. However, STI fluctuates with different combinations of clay, sand, and silt in the soil layers.

### Field experiments

Conventional tillage implements were used in this study. The utilized tillage implements were a duck foot type chisel plow, a disk plow, and a moldboard plow. All the investigated plows were attached to the three point linked hitch of the tractor. The characteristics of each plow are shown in Table 1.

**Table 1:** The characteristics of the investigated plows.

Items	Chisel plow	Moldboard plow	Disk plow
No. of shanks	9	3	3
Plow width, (cm)	225	105	105
Rake angle, (°)	22	–	–
Share width, (cm)	20	35	–
Disk diameter, (cm)	–	–	70
Disk tilt angle, (°)	–	–	15
Disk angle, (°)	–	–	42

The field trials were carried out in different soils of two experimental sites employing four intensities of plowing depths (10, 15, 20, and 25 cm), four levels of plowing speeds (3, 4, 5, and 6 km/h), and four intensities of moisture content of the soil (8.5, 12.5, 16.5, and 20.5%db). All plowing tests had three replications and average values were considered in the analysis as every plow had 128 data points (all data is 384 points, the 319 data points used for training purpose and 65 data points used for testing purpose). The test area was 75 × 2.5 m^2^ for each experiment. Preceding each field experiment, a 5 m buffer (run in) zone was set up to allow the tractor-plow unit achieve a stable state of plowing speed and depth before the actual run through the test area. The plowing speeds were quantified using the tractor hand throttle and constant gear ratio (monitored on the tractor’s dashboard). The plowing speed was determined by dividing specific distance over time. The plowing depths were selected and fixed using the tractor depth controller. The tillage depth was acquired using a steel measuring tape with the undisturbed soil surface as a reference.

A Furat tractor of 70 hp in 2WD mode was employed in this study. The inflation pressure of the tractor drive wheels was adjusted to 120 kPa. Slippage of the tractor drive wheels is commonly determined by quantifying two speeds. The first speed is no load speed, which denotes the speed of the tractor without pulling the plow, and the second speed is loading speed, which symbolizes the tillage speed using the following formula^16,17^:

2 $$ RWS = \left( {1 - \frac{{V_{a} }}{{V_{t} }}} \right) \times 100 $$

where RWS is tractor rear wheel slip (%), V_a_ is the tillage speed or loading speed (m/s), and V_t_ is no load speed or the speed of the tractor without pulling the plow (m/s).

### Multiple linear regression model (MLR)

Regression models describe the existing relationships between a set of dependent and independent variables especially in small sample sizes^18^. In this research, the MLR model is employed to model the relationship between tractor rear wheel slip and the operation variables for each plow. The MLR model assumes that the variable components are independent and may not match the actual situation^19^. The general MLR model is expressed as follows:

3 $$ Y = \beta_{0} + \beta_{1} x_{1} + \beta_{2} x_{2} + \beta_{3} x_{3} + \beta_{4} x_{4} + \cdots + \beta_{q} x_{q} $$

where Y is the dependent variable, observed values; x_1_, x_2_, x_3_, x_4,_ and x_q_ are the explanatory or independent variables, and $\beta_{0} ,\beta_{1} ,\beta_{2} ,\beta_{3} ,\beta_{4} ,\,{\text{and}}\,\beta_{q}$ are the multiple regression coefficients.

In Eq. (3), *Y* represents the tractor rear wheel slip as the dependent variable, and x_1_, x_2_, x_3_, and x_4_ are the set of predictor variables comprising plowing depth, soil moisture content, plowing speed, and soil texture index, respectively.

### Artificial neural network model

**Figure 1:**
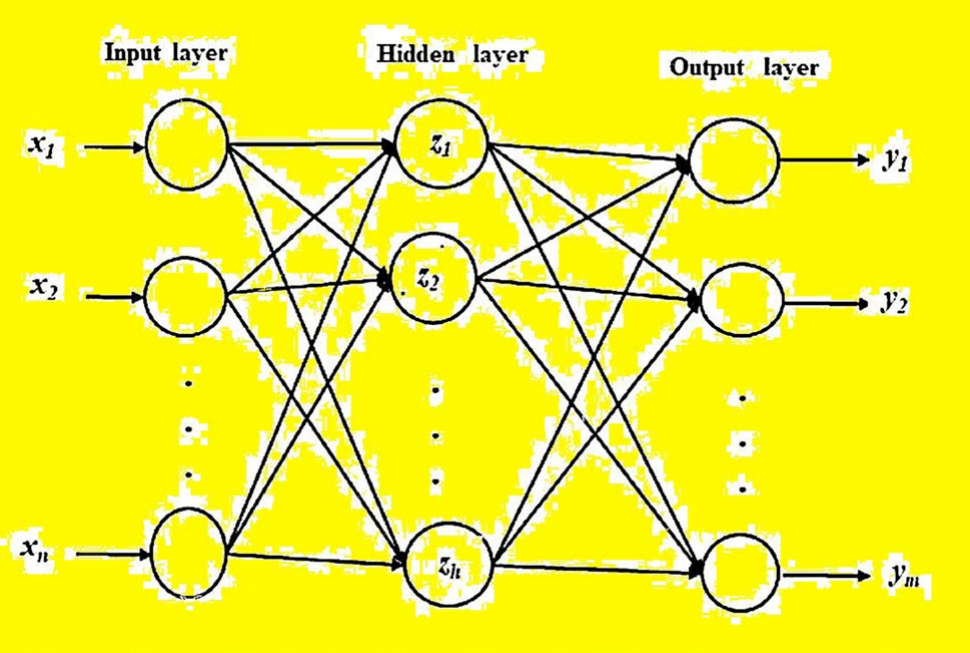
The general architecture of the ANN model.

An ANN is a nonlinear mathematical model capable of demonstrating randomly complex nonlinear relationship. It can be utilized to communicate inputs and outputs of any relationship^20^. The key advantage of such ANN model over statistical methods is that it does not need the data on complex relationships under examination to be clearly designated in mathematical terms^21^. The architecture of an ANN involves of a set of neurons (also called nodes) associated by weights and generally structured in different layers namely: input, hidden and output layers. Figure 1 depicts the ANN architecture with different input variables, *x*_1_ … *x*_*n*_.

Each neuron in a specific layer processes and takes the weighted input from a former layer and spreads its output to neuron(s) in the next layer through connection links. Each connection link is apportioned a weight, which denotes, in a numerical way, the connection link strength. The weighted summation of all inputs to a neuron is transformed to an output layer using an activation transfer function (typically a sigmoid function). Most ANNs have three or more layers: an input layer, which is engaged to present actual patterns to the specific network; an output layer, which is employed to yield a fitting response to the assumed inputs; and one or more hidden layers, which are used as a collection of feature predictors. Selection of suitable network topology of an ANN model is one of the important tasks for the ANN model creation process^21^. However, most of the network generally used is of the feed-forward ANN type. The structure of such topology has the benefit of being able to acquire complex nonlinear relationships between input and output patterns^22^.

Consider an ANN topology with *n* input nodes (*x*_1_, …, *x*_*n*_), *h* hidden nodes (*z*_1_, …, *z*_*h*_), and *m* output nodes (*y*_1_, …, *y*_*m*_), then let *i,j* and *k* be the keys expressive input, hidden, and output layers, respectively^21^. Also, let *τ*_*j*_ be the bias for node *z*_*j*_ and *φ*_*κ*_ be the bias for node *y*_*k*_. Let *w*_*ij*_ be the weight of the connection from node *x*_*i*_ to node *z*_*j*_ and *β*_*jκ*_ be the weight of connection from node *z*_*j*_ to *y*_*k*_. The summation function that an ANN calculates is:

4 $$ y_{k} = g_{A} \left( {\sum\limits_{j = 1}^{h} {z_{j} \beta_{jk} + \phi_{k} } } \right) $$

5 $$ z_{j} = f_{A} \left( {\sum\limits_{i = 1}^{n} {x_{i} w_{ij} + \tau_{j} } } \right) $$

where *g*_*A*_ and *f*_*A*_ are activation transfer functions, which have characteristics of non-decreasing, typically continuous, and bounded. The typical select is activation transfer belonged to sigmoid function for a variable *s* which is symbolized as:

6 $$ f(s)\;or\quad g(s) = \frac{1}{{1 + e^{ - s} }} $$

In developing a neural network model for a regression problem, the usual method is to establish the model based on the raw data, without any scaling of the input or output parameters. As a result, the model performance will be mostly poor^23^. So, data standardization or normalization is a common choice in training neural networks. However, a neural network model trained on standardized data produced better outcomes in general^24^. Moreover, the normalization of inputs and outputs also guarantees better results since relative values of inputs and outputs are normally different in more than one order of scale^25^.Thus, to develop our ANN model, the input and output values were normalized between 0.15 and 0.85 by the software, according to the following equation.

7 $$ T = \frac{{(t - t_{\min } )}}{{(t_{\max } - t_{\min } )}} \times (0.85 - 0.15) + 0.15 $$

here *T* is the normalized value, *t* symbolizes the actual values of the input and output variables; and *t*_*min*_ and* t*_*max*_ are the minimum and maximum values of input and output variables, respectively, in the training dataset. The dataset contained 128 points for each plow (total 384 points).

The training of an ANN model includes discovering the best weight vector for all connection links. Many training techniques are available^21^. To build the ANN model, we used a commercial neural network software called Qnet2000 under Windows^26^. Qnet2000 solves ANN problems based on a backpropagation training algorithm, and it can make predictions based on the input vector set by the researcher^27^.

**Figure 2:**
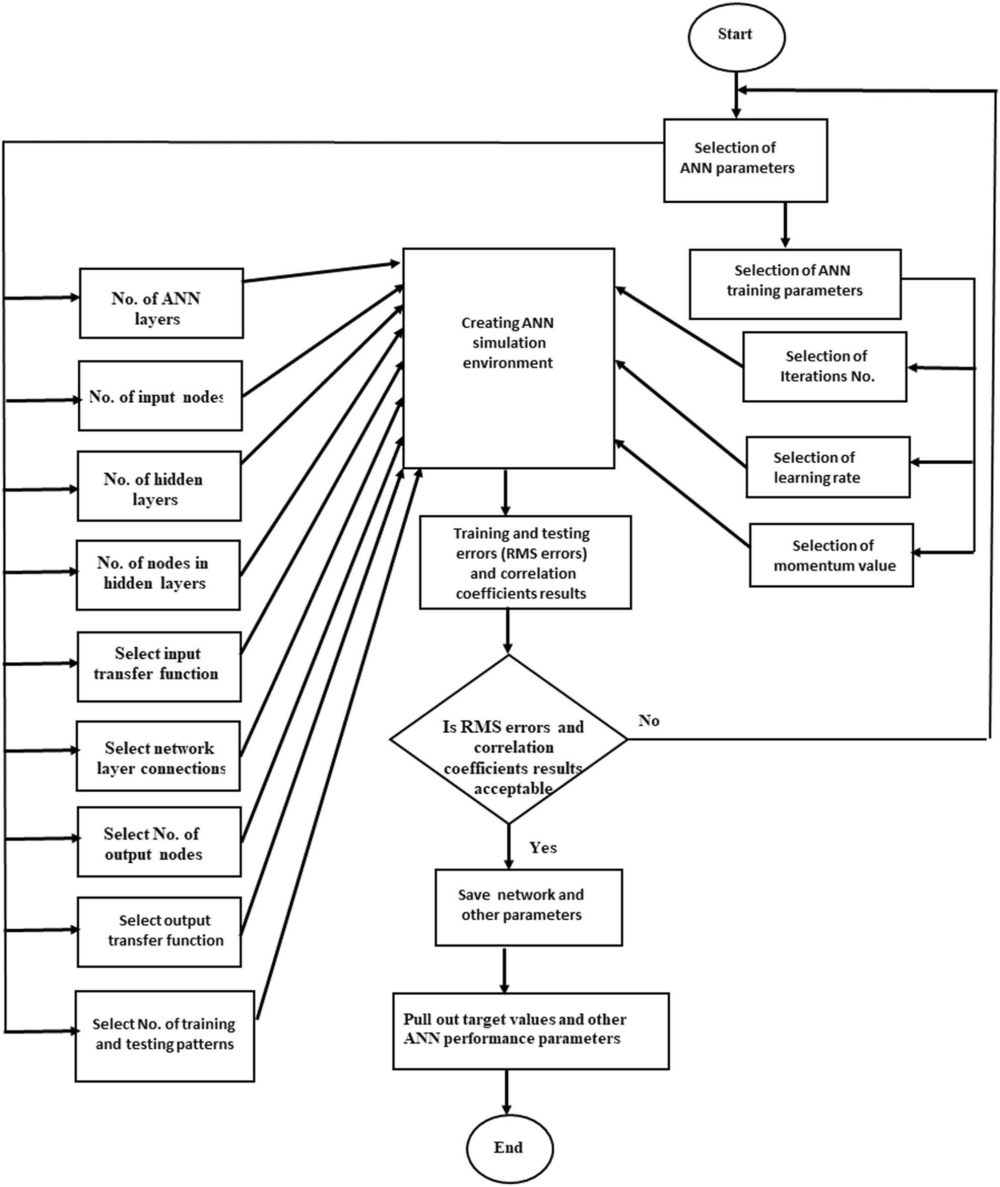
Qnet 2000 software procedures for developing an ANN model^29^.

The activated transfer function was sigmoid due to its superior performance, and it is generally employed in feed-forward neural-network models^28^. The dataset employed for developing the ANN model was separated into two datasets: training dataset and testing dataset. The software was randomly oriented for selecting testing data points of 65 patterns. A flowchart labeling the different solving stages for developing the ANN model using Qnet2000 is explained in Fig. 2^29^.

**Figure 3:**
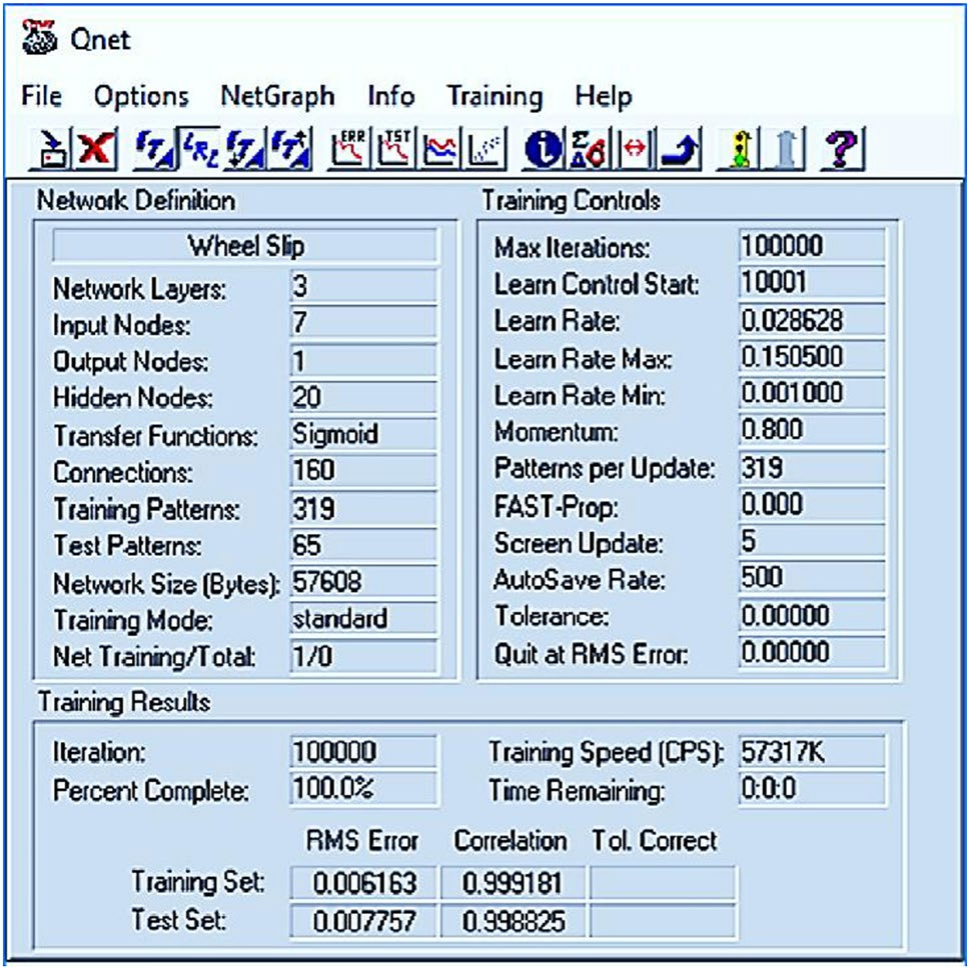
The definitions and training control parameters of the best ANN model acquired from Qnet 2000 software.

Overall, the quality of the established ANN is greatly dependable not only on ANN training procedure and its parameters but also on many ANN structural variables such as the number of hidden layers and neurons per layer which have to be selected during training stage and their values are very vital to the precision of an ANN model^30^. However, the incorporation of ANN and optimization tool offers a good method for selecting neural network structural variables or hyper parameter-tuning and improving the network performance using validation dataset^30^. Usually, the word “validation dataset” is used interchangeably with the word “test dataset” and denotes to a sample of the dataset held back from training the model^31^. In general, ANN structural variables or hyper parameter-tuning are commonly selected by previous knowledge in trial and error procedure^30^. Thus, during construction of the developed ANN model using feed forward structure based on an error backpropagation algorithm in this study, the trial-and-error procedure using only two datasets (training and testing) was used to select the type of activation function, number of hidden layers, number of neurons in hidden layers, learning rate, momentum factor, and number of epochs. We first started with one hidden layer and changed its number of neurons from 2 to 25 and the momentum factor was kept constant at 0.8. However, the learning rate was modified by the software, and the sigmoid activation function was selected due to its popularity in agricultural applications^28^. We next tested increasing number of hidden layers by 2 and 3 and the number of neurons. Different ANN structures were tested to reach the best configuration of ANN model and number of epochs was determined by looking at values of correlation coefficients and values of error of training and testing data, as shown in Fig. 3. The best ANN model had input, one hidden, and output layers.

The training stage was achieved by offering the network a set of field tillage data in an inputs-output pattern form. Each training pair includes an input set of seven parameters consisting of the chisel, moldboard, and disk plows, soil texture index, plowing speed, plowing depth, and soil moisture content. The corresponding output layer was comprised of the tractor rear wheel slip.

In the learning stage, the network is assessed by input of the parameters in the input vector for the first training pairs, followed by a correct calculation through the neurons in the hidden layer and prediction of the response output. The value of the training error between the desired and predicted tractor rear wheel slip is calculated for training and testing datasets and stored. The network is then offered with extra training patterns until it finishes all the existing data. Next, the training errors are calculated and backpropagated to the network. The weights (connection strength) and biases between neurons are modified during the backpropagation process to decrease the training error. At the end of the training stage, the neural network implemented is the one that gives the smallest training error with perfect correlation.

**Figure 4:**
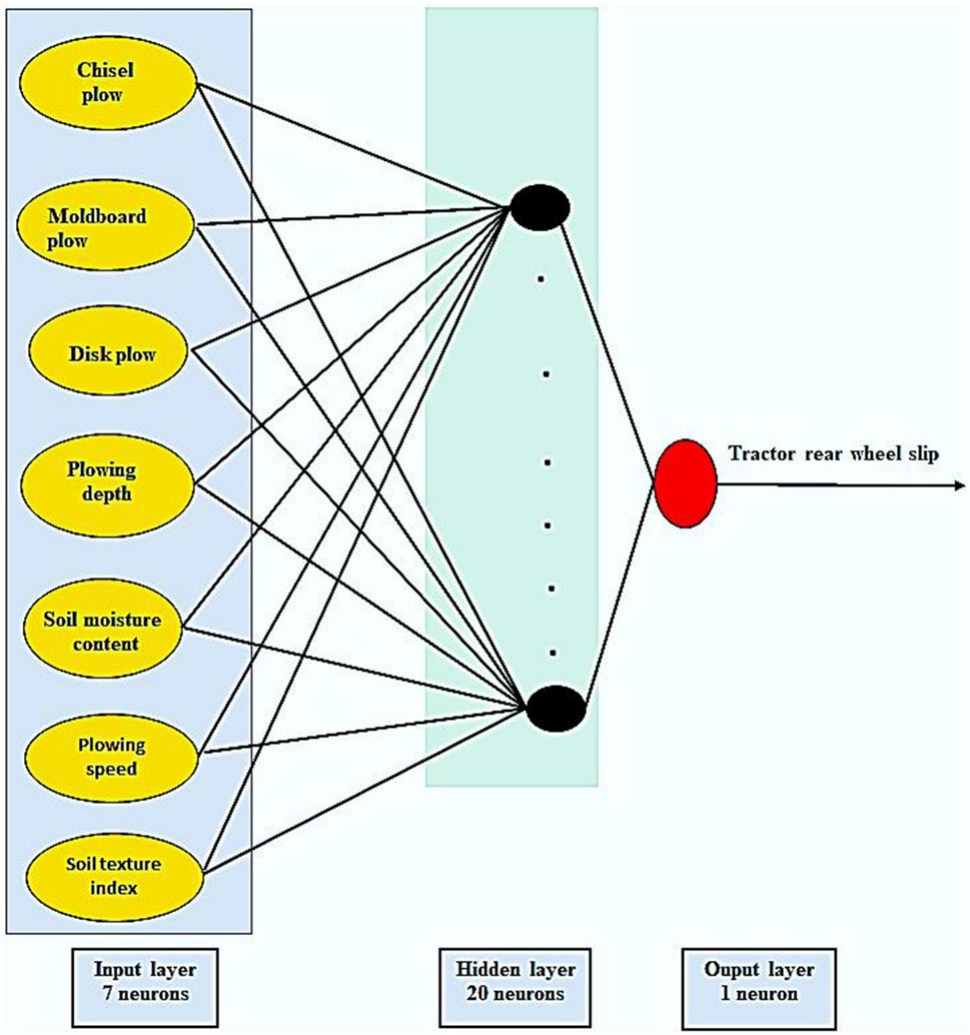
The resulting ANN model had 7 neurons for the input layer, 20 neurons for the hidden layer, and one neuron for the output layer.

After numerous attempts to changes the network structure and the numbers of nodes and hidden layers, we selected the best performing network consisting of 7 nodes in the input layer, 20 neurons in the hidden layer, and one neuron in the output layer (Fig. 4).

### Performance evaluation criteria

By matching the predictions to the actual values of tractor rear wheel slip in the testing dataset, a model trained on the training set can be assessed using multi-criteria methodologies. These criteria include the root mean squared error (RMSE) and the mean absolute error (MAE)^32^. Additionally, scatter plots are employed for visual assessment of the actual and predicted tractor rear wheel slip.

8 $$ RMSE = \sqrt {\frac{1}{n}\sum\nolimits_{i = 1}^{n} {\left( {P_{i} - O_{i} } \right)^{2} } } $$

9 $$ MAE = \frac{1}{n}\sum\nolimits_{i = 1}^{n} {\left| {P_{i} - O_{i} } \right|} $$

here *P*_*i*_ and *O*_*i*_ are the predicted and actual tractor rear wheel slip, respectively, and *n* is the total number of observations. Consequently, the prediction accuracy (PA) of the models is expressed using Eq. (10)^33^.

10 $$ PA = \left( {1 - \frac{1}{n}\sum\nolimits_{i = 1}^{n} {\frac{{\left| {O_{i} - P_{i} } \right|}}{{O_{i} }}} } \right) \times 100 $$

### Contribution and effect of the ANN model inputs on tractor rear wheel slip

After an ANN is near its fully trained state, it is often useful to determine what inputs are important to an ANN’s output response. For each output node, a contribution percent was produced showing the relative importance of each input on that particular output. Sensitivities are determined by cycling each input for all training patterns and computing the effect on the ANN’s output response. This method of computing sensitivity assumes that each input value is independent of all other inputs. The input node's minimum-average-maximum values, as determined by the training data, are used as the three values used in the cycling process. The output change for the (minimum-average) and (maximum-average) are then calculated and totaled in a root sum square calculation for each input node. Thus, after doing this for each input node and case, it is possible to compare the relative effect that each node has on the output response. The output node changes are then used to compute a simple percent effect for each input node as follows according to Vesta Services^26^:

11 $$ RE = \frac{RMSE\_C}{{RMSE\_T}} \times 100 $$

where* RE is* relative effect that each node (inputs) has on the output response (%).* RMSE_C* is root mean square error of output changes when node 1 for example is varied (means first input,* RMSE_T* is root mean square error of output changes for all input nodes (inputs).

## Results and discussion

### Analysis of the rear wheel slip values for an agricultural tractor in 2WD mode

By calculating soil text index (STI), it is clear that STI in the site I was 0.342 and in the site II, it was 0.573. However, Table 2 shows the statistical description of the tractor rear wheel slip during the tillage process as it was acquired from the two experimental sites. The higher coefficient of variation may be attributed to the high variation between low and high rear wheel slip values inside all data, but for each plow data, the coefficients of variation were in the range 14.12–16.99% for chisel plow, 14.46–16.99% for disk plow, and 17.97–21.03% for moldboard plow. Moreover, the higher value of the average tractor rear wheel slip was found at the site II (STI = 0.573) as compared to the site I (STI = 0.342) for all plows as set in Table 2. This was due to the fact that the wheel slip increased with the increase in draft force of the tractor^34^, and increasing of draft force was due to increasing STI^35,36^. Additionally, Janulevičius and Giedra^37^ showed the slippage and characteristics of traction were dependent on the parameters of the driving wheels, soil type, and the mechanical and physical properties of the soil. Finally, increasing soil texture index led to increases in tractor rear wheel slip in 2WD mode due to increasing maximum soil shear^38^.

**Table 2:** Statistical description of the tractor rear wheel slip during tillage process**.**

Statistical criteria	Chisel plow		Moldboard plow		Disk plow	
	Site I	Site II	Site I	Site II	Site I	Site II
Minimum, (%)	2.61	2.83	3.04	3.24	4.43	4.80
Maximum, (%)	14.65	15.14	19.00	19.37	19.36	19.56
Average, (%)	6.68	7.18	8.15	8.43	9.24	9.59
Median, (%)	6.24	6.93	7.54	7.76	8.36	8.68
Standard deviation, (%)	3.07	3.09	3.98	3.99	3.66	3.59
Coefficient of variation, (%)	45.88	43.10	48.77	47.30	39.58	37.40

**Figure 5:**
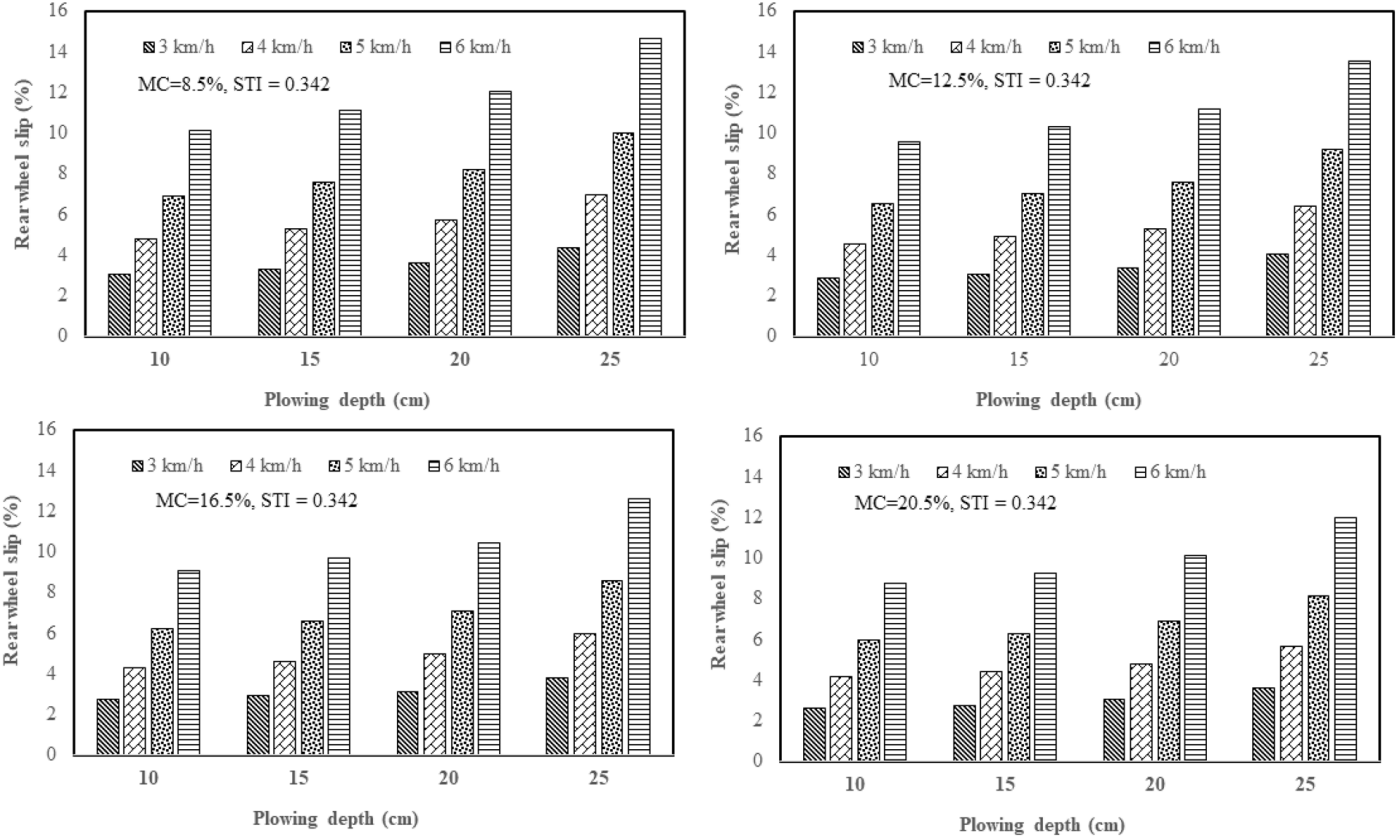
Effect of plowing speed and depth on the tractor rear wheel slip in 2WD mode at different levels of soil moisture content during plowing process by chisel plow in soil with a texture index of 0.342.

**Figure 6:**
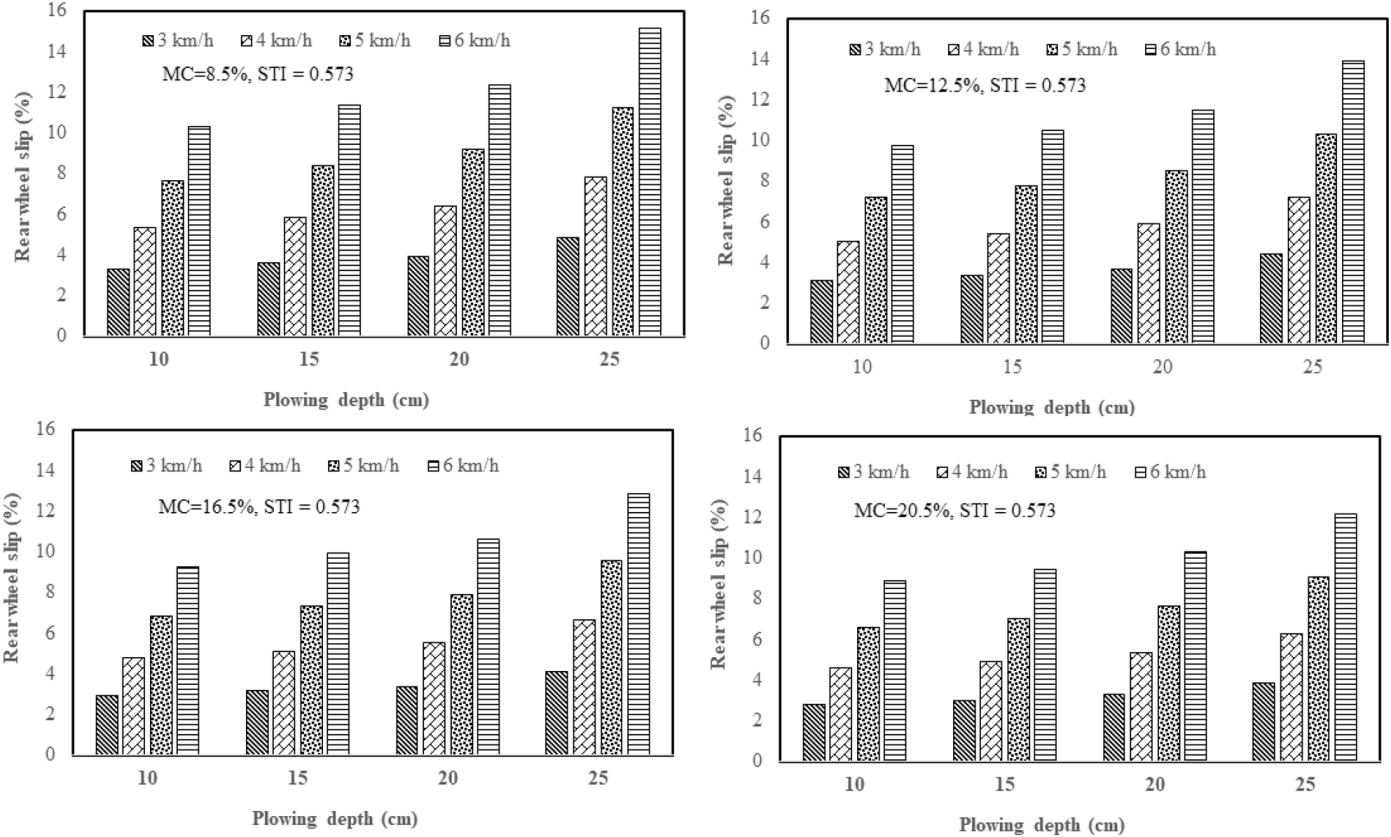
Effect of plowing speed and depth on the tractor rear wheel slip in 2WD mode at different levels of soil moisture content during plowing process by chisel plow in soil with a texture index of 0.573.

**Figure 7:**
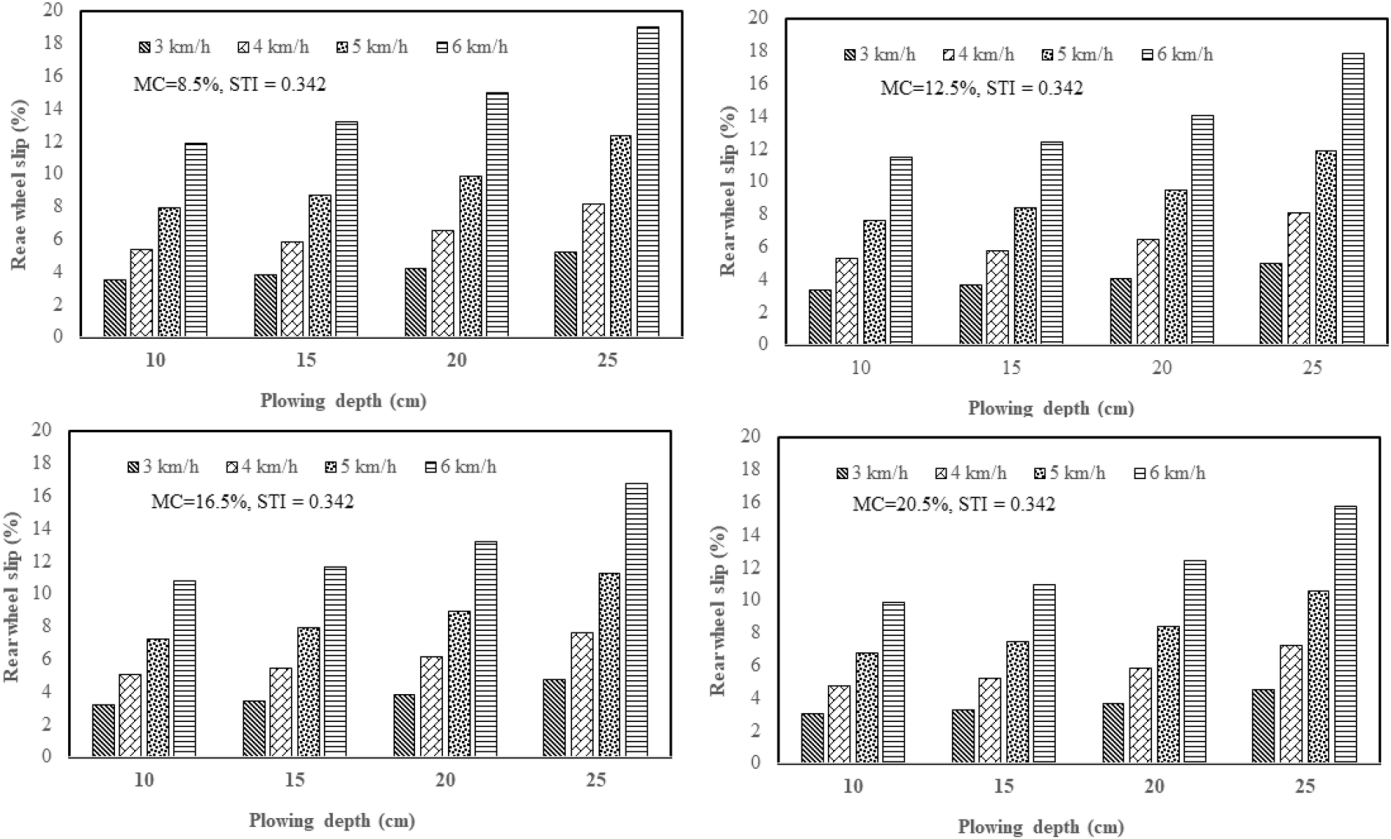
Effect of plowing speed and depth on the tractor rear wheel slip in 2WD mode at different levels of soil moisture content during plowing process by moldboard plow in soil with a texture index of 0.342.

**Figure 8:**
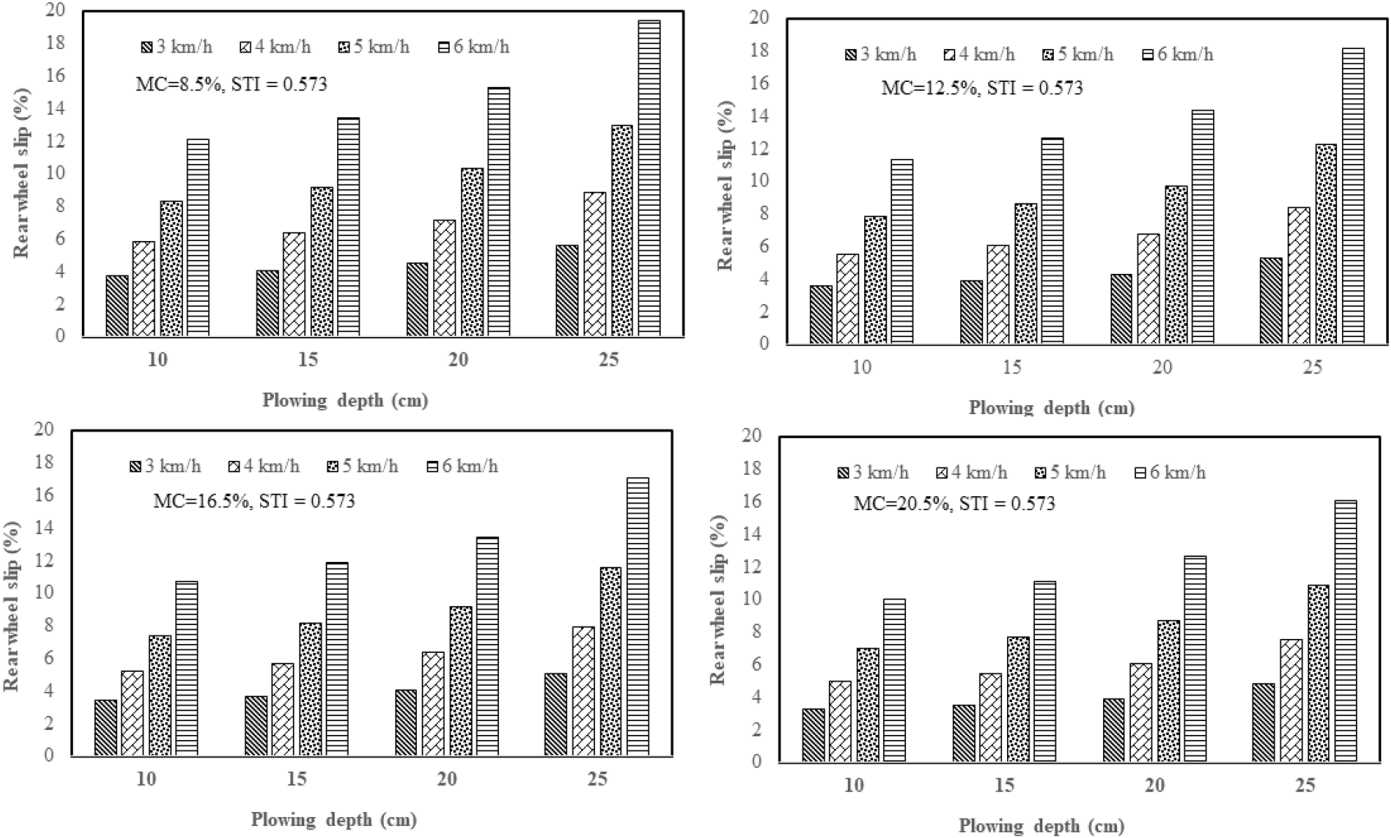
Effect of plowing speed and depth on the tractor rear wheel slip in 2WD mode at different levels of soil moisture content during plowing process by moldboard plow in soil with a texture index of 0.573.

**Figure 9:**
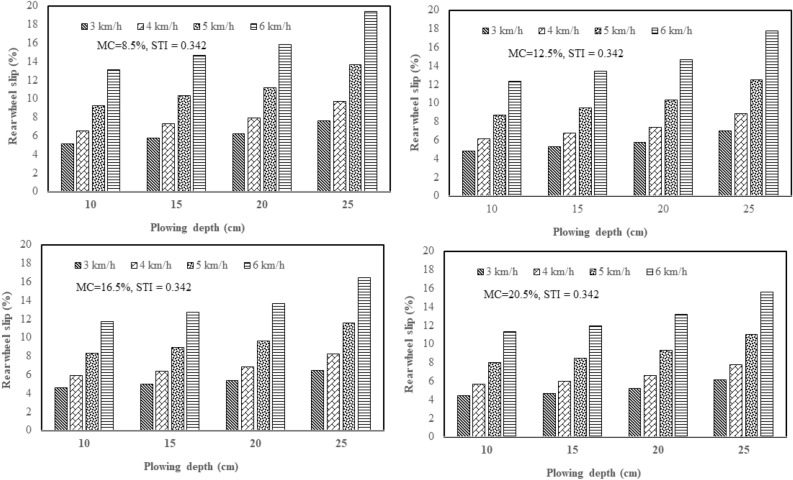
Effect of plowing speed and depth on the tractor rear wheel slip in 2WD mode at different levels of soil moisture content during plowing process by disk plow in soil with a texture index of 0.342.

**Figure 10:**
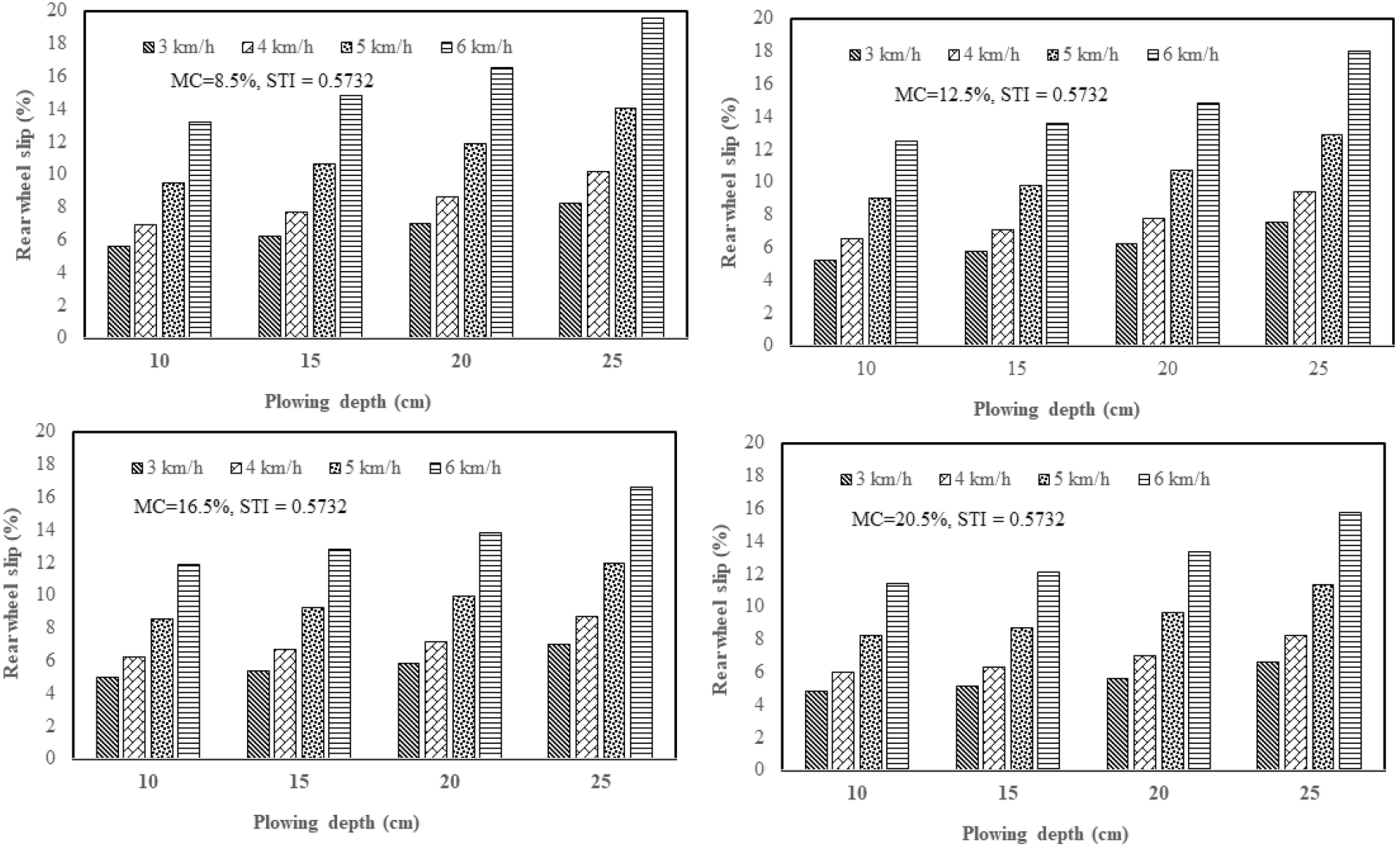
Effect of plowing speed and depth on the tractor rear wheel slip in 2WD mode at different levels of soil moisture content during plowing process by disk plow in soil with a texture index of 0.573.

From the results of this study, the effect of plowing speed and depth on the tractor rear wheel slip in 2WD mode was clarified with three plows: a chisel plow, a moldboard plow, and a disk plow, in the soil with texture indices of 0.342 and 0.573 and different soil moisture contents (4 levels of moisture). Figure 5 shows how plowing speed and depth affect the tractor rear wheel slip in 2WD mode with a chisel plow in soil with texture index of 0.342 and different soil moisture contents. Figure 6 shows how plowing speed and depth affect the tractor rear wheel slip in 2WD mode with a chisel plow in soil with texture index of 0.573 and different soil moisture contents. Figure 7 shows how plowing speed and depth affect the tractor rear wheel slip in 2WD mode with a moldboard plow in soil with texture index of 0.342 and different soil moisture contents. Figure 8 shows how plowing speed and depth affect the tractor rear wheel slip in 2WD mode with a moldboard plow in soil with texture index of 0.573 and different soil moisture contents. Figure 9 shows how plowing speed and depth affect the tractor rear wheel slip in 2WD mode with a disk plow in soil with texture index of 0.342 and different soil moisture contents. Figure 10 shows how plowing speed and depth affect the tractor rear wheel slip in 2WD mode with a disk plow in soil with texture index of 0.573 and different soil moisture contents.

The mentioned figures (Figs. 5, 6, 7, 8, 9 and 10) illustrate that slippage percentage is observed considerable rise with increase of plowing speed and tillage depth. This finding was agreed with those obtained by AL Aridhee et al.^39^, who observed significant increase in slippage percentage with changing of plowing speed from 4.2 to 6.5 km/h and with the changing of tillage depth from 15 to 20 cm. Additionally, in another study^40^, increasing rear wheels’ slip was detected due to increasing tillage depth and moisture content of the soil which resulted in a decrease of tractive efficiency of the tractor. Also, Almaliki et al.^8^ noted that tractor slippage using a chisel plow progressively increased with increasing tillage depth to a factor of 187% at a depth of 25 cm. This may be attributed to the increased drawbar pull at greater tillage depth. Increase of draft force of the plow led to higher required net traction which then led to a boost in the movement of soil particles. In addition, Almaliki et al.^8^ showed the trend of increasing slippage of tractor wheels with increasing plowing speed. They noted a speed increase from 0.54 to 1.53 m/s created a major increase in slippage of approximately 116%. This relates back to increasing drawbar pull for a moving plow with incremental increases in plowing speed. Hence, horizontal force produced by the tractor may be insufficient and as an end, the slippage increases. Moreover, Alnajjar^38^ showed that the wheel slip in 2WD mode for an agricultural tractor increased exponentially as plowing speed increased. Furthermore, it is clear from figures that the slippage percentage considerable increased with the decreasing of the moisture content of the soil at constant levels of both plowing depth and speed at each site and this may be due to increasing soil moisture content will decrease cohesion value between tractor tire and the soil as well as increase internal soil friction angle^38^ since Huang et al.^41^ reported that the main influence of increasing soil water content on shear strength is decreasing the cohesion.

The range of tractor rear wheel slip for chisel plow using different plowing speeds, plowing depths with different moisture content of the soil in the first site (STI = 0.342) was 2.61% to 14.65%, and in the second experimental site (STI = 0.573), it was 2.83% to 15.14% (Table 2). Under the same conditions, the range for moldboard plow was 3.04% to 19.00% in the first site and 3.24% to 19.37% in the second site. Results using a disk plow showed the slip range was 4.43% to 19.36% in the first site and 4.80% to 19.56% in the second site.

In this study, inverse relationship between tractor rear wheel slip and soil moisture content was reported as example, at 10 cm plowing depth and at 6 km/h plowing speed, the increasing soil moisture content from 8.5 to 20.5%, the tractor rear wheel slip decreased from 12.10% to 10.02% when the moldboard plow was used as shown in Fig. 8 for site II (STI = 0.573) and this trend was proved by Mamkagh^42^, who reported that the soil moisture content increased from 7 to 15%, the tractor rear wheel slip decreased from 20 to 16% when the moldboard plow was used. However, in other research studies^2,43^, increasing soil moisture content led to increase tractor rear wheel slip, however, the different results may be due to working characteristics change like tillage speed, tillage depth and soil structure^44^.

### Wheel slip ANN and regression models analysis

#### Regression model

An Excel spread sheet was used to obtain the MLR regression coefficients using the same training data used for ANN. The independent parameters included in the MLR model were the moisture content of the soil, plowing speed, plowing depth, soil texture index. The coefficients of determination, number of processing data, prediction accuracy, and multiple linear regression coefficients for each type of plow are shown in Table 3. In addition, Eqs. (12)–(14) show the linear regression equations obtained from the experimental data to get tractor rear wheel slip (RWS, %) for chisel plow, moldboard plow, and disk plow, respectively.

12 $$ RWS = - 6.594 + 0.163 \times PD - 0.109 \times MC + 2.537 \times PS + 1.924 \times STI $$

13 $$ RWS = - 8.718 + 0.226 \times PD - 0.092 \times MC + 3.045 \times PS + 1.378 \times STI $$

14 $$ RWS = - 6.236 + 0.227 \times PD - 0.161 \times MC + 2.909 \times PS + 2.034 \times STI $$

**Table 3:** Regression coefficients for rear wheel slip of a tractor during tillage process using different tillage implements.

Variables	Regression coefficients symbol	Regression coefficients for rear wheel slip of a tractor		
		Chisel plow	Moldboard plow	Disk plow
Intercept	$\beta_{0}$	− 6.594	− 8.718	− 6.236
Plowing depth (PD, cm)	$\beta_{1}$	0.163	0.226	0.227
Soil moisture content (MC, %db)	$\beta_{2}$	− 0.109	− 0.092	− 0.161
Plowing speed (PS, km/h)	$\beta_{3}$	2.537	3.045	2.909
Soil texture index (STI, –)	$\beta_{4}$	1.924	1.378	2.034
Coefficient of determination (R^2^)	0.962	0.945	0.938	
Prediction accuracy, (%)	91.83	90.03	91.48	
No. of processing data	110	102	107	

**Figure 11:**
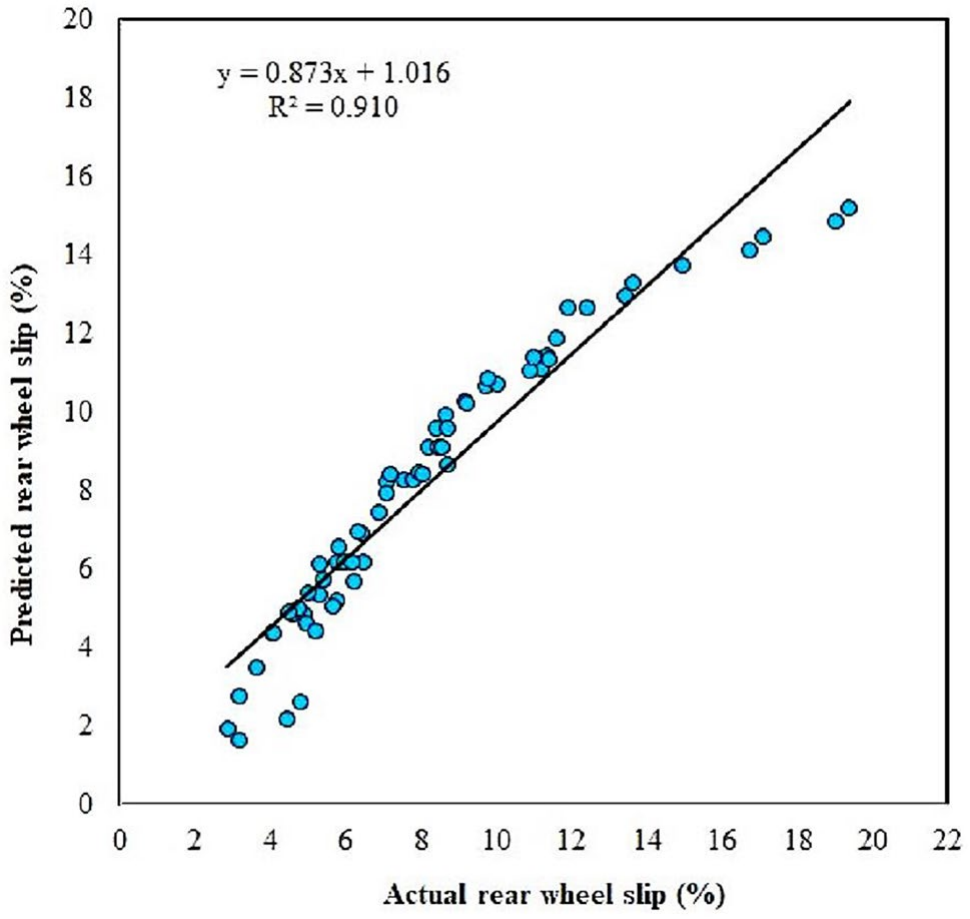
Variations of the observed and MLR-predicted tractor rear wheel slip during tillage operation using the testing data set.

The result for the prediction of RWS employing the operation parameters using MLR are presented in Fig. 11. Using the testing data set, it is observed that the optimal prediction of RWS using MLR was obtained by incorporating all the input parameters, resulting in *R*^*2*^ of 0.910. The results in Table 3 show that the combination of operation parameters predicted RWS with a prediction accuracy of 91.83%, 90.03%, and 91.48% for chisel, moldboard, and disk plow, respectively. Additionally, Table 3 shows that plowing speed had the highest impact on RWS prediction, followed by soil texture index. Also, there is an indication of RWS increasing with plowing speed, plowing depth, and soil texture index. Meanwhile, there is an indication of RWS decreasing with soil moisture content.

#### ANN model

**Figure 12:**
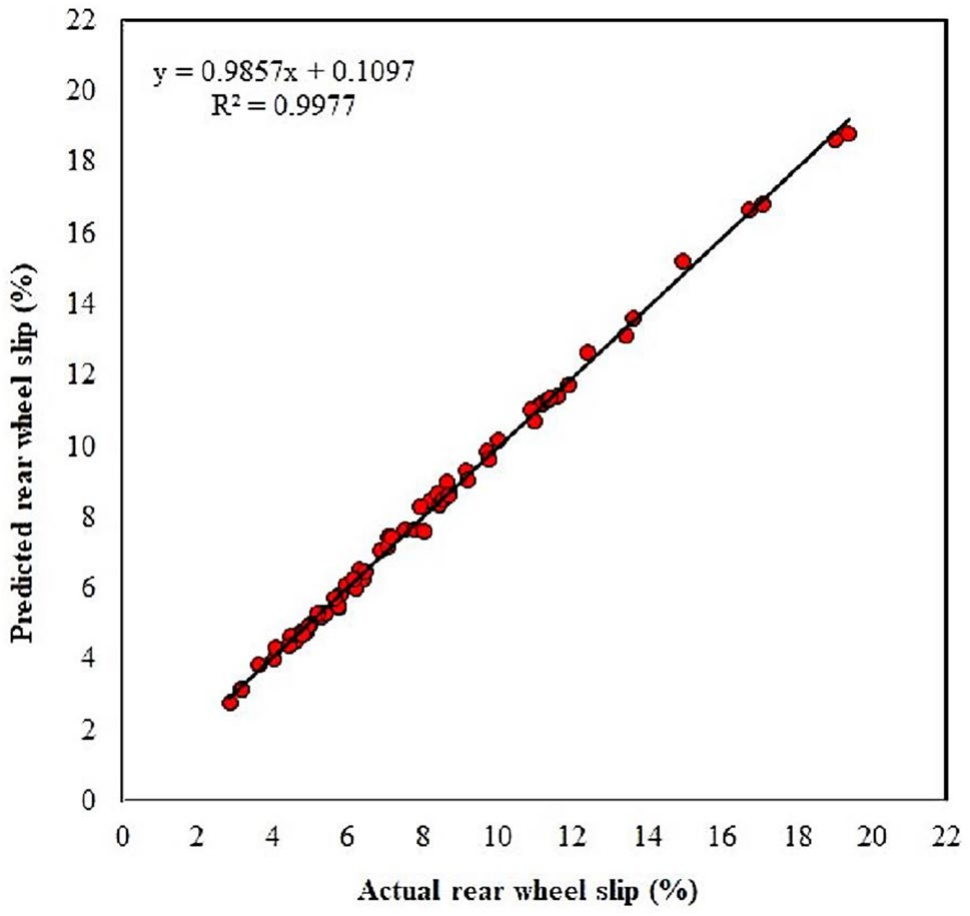
Variations of the observed and ANN-predicted tractor rear wheel slip during tillage operation using the testing data set.

The tractor rear wheel slip was modeled using ANN. Results showed that the ANN model with the 7-20-1 structure and standard backpropagation training algorithm performed well. The coefficients of correlation during the training and testing phases were dramatically altered and improved with each iteration and recorded values of 0.999181 and 0.998825 during the training and testing phases, respectively, as shown in Fig. 3. In addition, the training algorithm was terminated when the training error had finished the epochs, the small error occurred, and the training error and testing error showed similar character of 0.006163% and 0.007757%, respectively (Fig. 3). Moreover, Fig. 12 is a chart showing the line of best fit between the actual tractor rear wheel slip and the values predicted by the network. The network developed with one hidden layer of 20 neurons had the highest slope, lowest ordinate, and highest correlation coefficient with the values of 0.9857, 0.1097, and 0.9977, respectively. The line alignment close to the data displays better fitting and more precision for the prediction of the network output using the testing dataset. The coefficient of determination (Fig. 12) indicates a high level of agreement between the experimental and predicted wheel slip results.

#### Comparison between prediction skill of the MLR and ANN models

**Figure 13:**
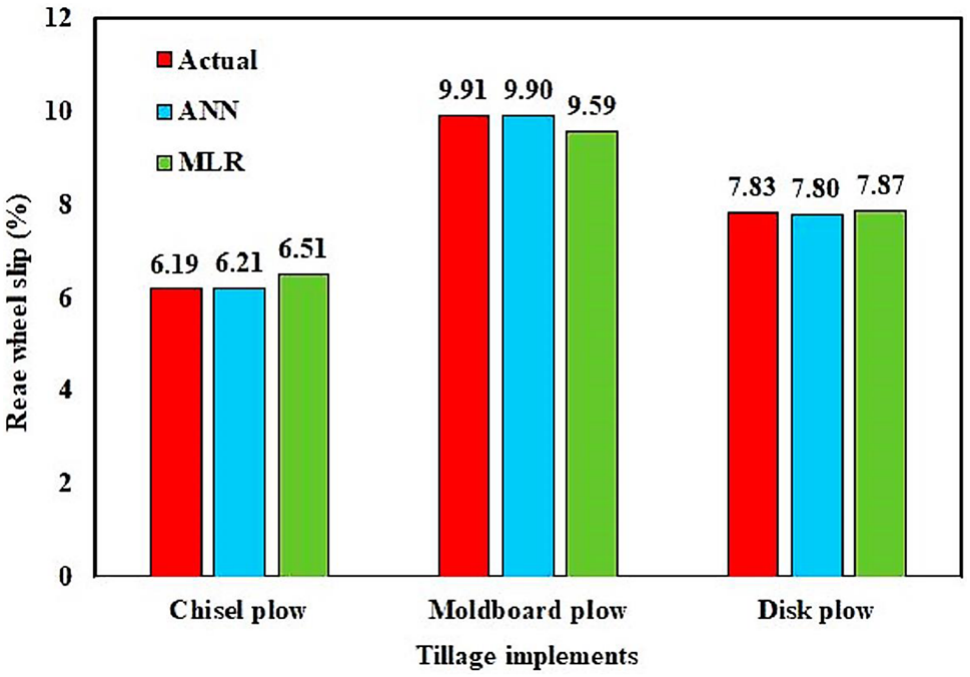
The average rear wheel slip from the ANN and MLR models compared to actual values of the three tillage implements using testing dataset.

To evaluate the performance of ANN model in relation to the MLR model, the average tractor rear wheel slip of the three tillage implements from the two models were compared using the testing dataset as described in Fig. 13. The comparison of the results gained from the ANN model and MLR model to predict the tractor rear wheel slip of the investigated plows indicated that the predicted data by those two models were close. The ANN model offered predicted data which was closer to the actual data compared to the results acquired from MLR model.

Table 4 displays values representing performance behavior of the ANN and MLR models. For chisel plow the ANN model with statistical performance criteria of R^2^ = 0.995, RMSE = 0.152%, and MAE = 0.118% implemented better than the MLR model with statistical performance criteria of R^2^ = 0.967, RMSE = 0.570%, and MAE = 0.468%. For moldboard plow, with statistical performance criteria of R^2^ = 0.998, RMSE = 0.238%, and MAE = 0.195%, the ANN model performed better than the MLR model which showed statistical performance criteria of R^2^ = 0.919, RMSE = 1.514%, and MAE = 1.018%. Finally, for disk plow, the ANN model with statistical performance criteria of R^2^ = 0.997, RMSE = 0.139%, and MAE = 0.119% performed better than the MLR model with statistical performance criteria of R^2^ = 0.929, RMSE = 0.944%, and MAE = 0.751%. The coefficient of determination (R^2^) and prediction accuracy (PA) for the MLR models were in the range of 0.929 to 0.967 and 88.36% to 91.32%, respectively which were lower than the R^2^ and PA of the ANN model, which were in the range of 0.995–0.998 and 97.86–98.34%, respectively (Table 4). The values of R^2^ of the investigated models were higher than 0.90, indicating that the correlations of the predicted rear wheel slip from the ANN and MLR models compared to actual values were very strong. However, R^2^ is usually used for the assessment of the goodness of linear fit of regression model in ANNs^45^ and the PA, RMSE, and MAE are employed to evaluate the errors of the predictions^46^. In a similar study, Shafaei et al.^13^ noticed that using an ANN model for estimating the wheel slip for moldboard plow, the best ANN topology had one hidden layer with 6 neurons, 658 training cycles, and input and output transfer function of tangent and Gaussian, respectively. The statistical performance calculated criteria for prediction of the wheel slip of the best ANN topology was R^2^ = 0.856 and RMSE = 3.026using their testing dataset. The soil had clay loam soil texture (i.e., 35% sand, 35% clay, and 30% silt) and flat topography. The mean moisture content and bulk density of the soil were 8.84 db% and 1.23 g/cm^3^. In their study, the precise relationship between tractor rear wheel slip and independent parameters was attained by the ANN model. Therefore, the ANN model can extract complicated relationships between output and input parameters, and as the ANN model trains itself, it can predict target based on multiple input parameters directly without expert knowledge. In several previous agricultural engineering research experiments, Abbaspour-Gilandeh et al.^33^ noted that reasonable accuracy of the ANN model can be expected versus the MLR model.

**Table 4:** Comparison between the calculated performance criteria for ANN and MLR models for prediction of tractor rear wheel slip during plowing process using testing dataset.

Tillage implements	ANN	ANN	ANN	ANN	MLR	MLR	MLR	MLR	No. of data points
	RMSE (%)	MAE (%)	R^2^	PA (%)	RMSE (%)	MAE (%)	R^2^ (–)	PA (%)	
Chisel plow	0.152	0.118	0.995	97.96	0.570	0.468	0.967	91.32	18
Moldboard plow	0.238	0.195	0.998	97.86	1.514	1.018	0.919	89.83	26
Disk plow	0.139	0.119	0.997	98.34	0.944	0.751	0.929	88.36	21

### Contribution of the ANN model inputs and their impact on the tractor rear wheel slip

This analysis displays the impact of the change of each independent variable on the ANNs prediction model. The contribution percentage of the four input variables and tillage implements to their output was considered using the established ANN model, and the results are illustrated in Table 5. This table can be used to define the relative contributions and importance of each parameter involved in the study. Table 5 shows that the contribution of the plowing speed on RWS prediction was the highest as compared to the other parameters, with a contribution percentage of 36.42%. Thus, plowing speed must be measured carefully for determining tractor rear wheel slip as one of the most important variables that characterize a wheeled tractor’s traction coupling properties is its slip when operating in a particular machine-tractor unit^47^. Ranjbarian et al.^48^ found that the slippage increased as forward velocity increased. Also, Abbaspour-Gilandeh et al.^33^ clarified the response of RWS of a tractor to increasing speed. ALkhafaji et al.^49^ indicated that, the speed factor has a significant effect on the percentage of slippage. As plowing speed increased, the slippage percentage increased. This was the result of the increase in the power required to break the soil and the impact speed that occurred between the soil slices and shanks also increased. Those two factors increased the actual time required to perform the work relative to the theoretical time which in turn resulted in a higher percentage of tractor slippage. Furthermore, compared to other plows, the chisel plow gave a higher contribution to slippage of the driving tractor wheels at 18.78% as indicated in Table 5. This may be attributed to the higher width of chisel plow^50^ or the higher required draft force of chisel plow^14^ .

**Table 5:** Contribution percentage of independent variables used in the ANN model for prediction of tractor rear wheel slip during tillage process.

Input node	Percent contribution
Chisel plow	18.78
Moldboard plow	13.64
Disk plow	9.80
Plowing depth	13.45
Soil moisture content	6.31
Plowing speed	36.42
Soil texture index	1.59

## Conclusions

This investigation presents an artificial neural network (ANN) model for predicting tractor rear wheel slip during the plowing process under different field conditions. Commercial ANN software was applied to develop a powerful and reliable model. On the basis of statistical performance criteria, the findings indicate that plowing speed, with a contribution value of 36.42%, had the strongest effect on tractor rear wheel slip of all the investigated variables. However, compared to other plows, the chisel plow gave a higher contribution to slippage of the driving tractor wheels at 18.78%. Ultimately, the ANN model produces precise and consistent results and can be effective in predicting tractor rear wheel slip.

## Data Availability

All data are shown within the article’s figures and tables.
